# Towards an Accessible Use of a Brain-Computer Interfaces-Based Home Care System through a Smartphone

**DOI:** 10.1155/2020/1843269

**Published:** 2020-08-28

**Authors:** Koun-Tem Sun, Kai-Lung Hsieh, Syuan-Rong Syu

**Affiliations:** Department of Information and Learning Technology, National University of Tainan, 33, Sec. 2, Shu-Lin St., Tainan 70005, Taiwan

## Abstract

This study proposes a home care system (HCS) based on a brain-computer interface (BCI) with a smartphone. The HCS provides daily help to motor-disabled people when a caregiver is not present. The aim of the study is two-fold: (1) to develop a BCI-based home care system to help end-users control their household appliances, and (2) to assess whether the architecture of the HCS is easy for motor-disabled people to use. A motion-strip is used to evoke event-related potentials (ERPs) in the brain of the user, and the system immediately processes these potentials to decode the user's intentions. The system, then, translates these intentions into application commands and sends them via Bluetooth to the user's smartphone to make an emergency call or to execute the corresponding app to emit an infrared (IR) signal to control a household appliance. Fifteen healthy and seven motor-disabled subjects (including the one with ALS) participated in the experiment. The average online accuracy was 81.8% and 78.1%, respectively. Using component N2P3 to discriminate targets from nontargets can increase the efficiency of the system. Results showed that the system allows end-users to use smartphone apps as long as they are using their brain waves. More important, only one electrode O1 is required to measure EEG signals, giving the system good practical usability. The HCS can, thus, improve the autonomy and self-reliance of its end-users.

## 1. Introduction

Individuals with locked-in syndrome (LIS), amyotrophic lateral sclerosis (ALS), spinal cord injury, and congenital or accidental nerve injury may experience serious obstacles in developing motor skills in their limbs, yet most of them have normal brain function [1–3]. When they cannot speak clearly, the demands they are trying to articulate cannot be understood [1, 4]. Because of the gradual loss of mobility, such individuals may need a 24-hour personal caregiver [5, 6]. A brain-computer interface (BCI) system would be practical for this population. A BCI can permit them to control external devices and enable them to perform some tasks by themselves [7–10].

Locked-in syndrome (LIS) is a condition in which a patient is aware but cannot communicate verbally or move because of complete paralysis of nearly all voluntary muscles in the body except for vertical eye movements and blinking [11]. It is caused by damage to specific portions of the lower brain and brainstem, with no harm to the upper brain. Thus, such patients are fully awake and alert and are aware not only of their abnormal situation but also, and to a full extent, of their surroundings. There are three categories of LIS: classic LIS, incomplete LIS, and total LIS [4, 12]. In total LIS, even the eyes are paralyzed [13].

ALS is a relatively rare neurodegenerative disorder characterized by gradual loss of both upper and lower motor neurons in the brain, brainstem, and spinal cord [14]. ALS usually starts at around the age of 60 and, in inherited cases, around the age of 50 [2]. The average survival from onset to death is 3 to 5 years [15]. Most people with classic LIS, incomplete LIS, or ALS are free to move their eyeballs. If the brain activity of these people is not affected and their eyeballs are free to move, then a BCI system can help them communicate with others [7, 16–18].

A BCI system is a system that connects the human brain and its surroundings. It enables people to communicate with others using their brain waves without muscle movement [9, 17, 19–22]. There are different technologies for measuring brain activity. Among these, electroencephalographs (EEG) are the most frequently used because of their many advantages, including lower cost, better portability, and higher temporal resolution [23]. Over the past two decades, there has been a dramatic proliferation of research concerned with a noninvasive/stimulus-driven/visual BCI (vBCI) system [23–25]. Such BCI systems obtain the user's brain potentials on the surface of the cortex via an EEG [7, 21, 24].

There are four different types of EEG-based BCI modalities: event-related desynchronization/synchronization (ERD/ERS), steady-state visual evoked potentials (SSVEP), event-related potentials (ERP), and slow cortical potentials (SCP). Among these, ERP and SSVEP-based BCIs are more practical than others because they support large numbers of output commands and need little training time [7].

Table 1 shows recent studies of BCI-based systems implemented in real-world scenarios.

Table 1 shows that applications of BCI systems include speller, robot control, healthcare, environmental control, and social network use. The graphic user interface (GUI) is roughly divided into the row-column (RC) paradigm, as with the speller system, and the direction paradigm, as with robot control. There are two broad categories of stimulation for ERP: flashing LED-light and motion-onset. Most studies use more than six electrodes to collect the user's brainwaves, and their average accuracy is good. However, only healthy subjects participated in these experiments. The home care system (HCS) in this study is an environmental control system. The arrangement of the options in the GUI is derived from the row-column paradigm, but its stimulation is motion-onset [25]. Based on our previous work [8], this GUI with motion-onset stimulation can significantly improve the target detection performance to achieve higher accuracy and shorten the stimulation time, in contrast to the stimulus intensification pattern used in the conventional P300-based system.

Event-related potentials (ERP), proposed by Sutton in 1965, are a series of potentials of a user's brain waves elicited by external stimuli. These potentials are time-dependent voltage fluctuations triggered by specific physical or psychological events [27]. An ERP-based vBCI system usually flashes a specific stimulus (such as text, pictures, or a flickering strip) on the graphic user interface (GUI) many times, and the user's brain waves respond to each stimulus [7, 9, 10, 28, 29]. An ERP-based vBCI system uses an EEG to obtain the users' brain rhythm and learn the basics of their brain system [30]. The EEG device amplifies and records the potentials of the user's brain waves [31, 32] and sends these signals to the vBCI system to classify and to interpret the specific features of the ERP components. Significant ERPs may, then, be extracted from the EEG by filtering and signal averaging methods [33]. In the final step, the BCI system converts these signals into instructions and outputs them [25, 34]. In this study, we use Ag/AgCl electrodes to record the weak potential of brain waves on the user's scalp. The system uses a 0.3∼15 Hz band-pass filter to filter the signals [35]. After multiple stimulations, the EEG signals are, then, superimposed and averaged to form the results of the ERP for each trial [8].

**Figure 1:**
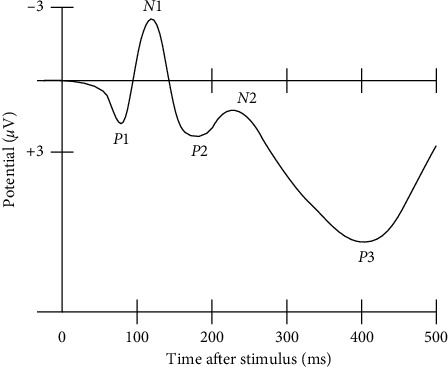
A waveform showing several ERP components, including the N200 (labelled N2) and P300 (labelled P3). Note that the ERP is plotted with negative voltages at the top, a common, but not universal, practice in ERP research [38].

ERP research provides an impersonal and workable discrimination method for a BCI system [36, 37]. This study adopts an ERP paradigm that combines oddball presentation and motion onset. This paradigm primarily exploits two ERP components, N200 and P300, instead of only P300 [25]. N200 (N2) and P300 (P3) are brain responses to specific cognitive tasks, as shown in Figure 1. A P300 peak in an ERP is a higher positive deflection of an event-related potential component and usually occurs around 300 ms after the target stimulus presentation [21, 28, 35, 39–42]. Conversely, an N200 trough is the lower negative deflection of event-related potential, and usually occurs nearly 200 ms after the target stimulus presentation [25, 39]. The N200 and P300 waves only occur if the subject is actively engaged in the task of detecting the targets [40, 43] and the waveform of the component P300 (N200) of the target stimulus is higher (lower) than that of the nontarget stimuli [10]. The amplitude of P300 (N200) depends on the improbability of the target stimulus. The latency of ERP components varies with the difficulty of discriminating between the target and nontarget stimuli [22, 35].

However, conventional BCIs have not become practical because they lack high accuracy and reliability and have low information transfer rate and user acceptability [44]. First, in a visual BCI system, although gaze is not a requirement [45], the presence of the gaze in a visual ERP-based BCI improves its performance. Thus, in the system, we prefer that the user stares at the GUI to select an option. If the users have lost their sight or cannot stare at the screen, it is more appropriate to use an auditory BCI [46] or the BCI system presented in the work of Thurlings et al. [45].

A flashing stimulation paradigm such as that the row-col paradigm [9, 22] easily leads to user eye fatigue. The motion-strip stimulation paradigm is more comfortable for the user's eyes because of the low luminance and low contrast required by the stimuli [25]. Furthermore, the identification rate and the accuracy of the motion-strip stimulation paradigm are better than that of the flashing stimulation paradigm [8, 25].

Second, the fewer the electrodes are, the more comfortable the user is. Based on the previous research results of our laboratory, when the user stares at the GUI of a vBCI system, the ERPs acquired from electrode O1 or O2 in the occipital area of the skull (the visual region of the human brain) can achieve a statistically significant difference between the target and nontarget stimuli [8, 26]. Thus, a system using only one electrode can obtain outstanding accuracy.

Third, the waveform and the amplitude of ERP (N200 and P300) of the target stimulus vary from person to person. Thus, the BCI system needs to use a more stable ERP component to increase its accuracy. Based on the results of our previous experiments, the accuracy from using component N2P3 is significantly higher and steadier than the accuracy obtained using any other ERP component. Herein, the N2P3 value of one option is the potential value (*μ*V) of its P300 minus that of its N200 [8, 26]. Thus, the system uses the N2P3 component online to expedite the experimental procedures. Moreover, users receive better real-time feedback. Yet, to test and verify that N2P3 is the most useful component for interpretation, we compared the ERP components (N200, P300, and N2P3) of the target option with those of nontarget options in an offline analysis.

According to the work of Huggins et al., if caregivers are absent, BCI users may want to perform tasks such as controlling the room temperature and lights or make emergency calls by themselves and feel more comfortable than they would using text communication [47]. However, such BCI systems are rare.

Because of the popularity of smartphones, several studies have applied BCI systems to control smartphones. Most of these studies explore merely dialing numbers [48, 49], accepting incoming calls [50], or calling contacts [49, 51]. Martínez-Cagigal et al. present a BCI system for controlling the social networking features of a smartphone [22]. Jayabhavani et al. pioneered a system which allows users to control wheelchairs, an approach that relates more closely to the topic of this paper [52].

This study develops a BCI-based home care system (HCS). The HCS allows the end-users to control their household appliances by themselves. Thus, end-users can reduce their dependence on the caregivers. In this study, there are two functions of a smartphone: to make an emergency call and to act as an adjustable infrared- (IR-) band remote controller. Thus, the user's smartphone must have or install an IR transmitter first. The corresponding app in the user's smartphone can, then, emit an IR signal to control the required household appliance.

Today, short-distance remote controls for devices in daily life make wide use of IR [53–55]. IR, sometimes called infrared light, is an electromagnetic radiation (EMR) with wavelengths longer than those of visible light. IR cannot pass through a wall. Thus, in the adjoining room, remote controls using the same IR wavelength do not interfere with each other. By contrast, in a chamber, the IR wavelength of all appliances should be distinct from each other. Therefore, each household appliance has a dedicated remote control.

Improving the personal autonomy and the self-reliance of end-users and giving them the ability to communicate with others are two of the primary missions of the HCS. Since the assessment of BCI systems with end-users is essential for ensuring a fair evaluation [22], we invited disabled users to test the system to learn whether the HCS is useful for motor-disabled people. Motor-disabled people have participated in some tests of BCI systems. The BCI system allows such individuals to perform actions with their brainwave signals [10, 22, 56]. For example, these patients can now spell out words with a BCI-speller [8, 16, 25, 57]. However, if the caregiver does not immediately notice the text on the screen, it will not be possible to help the patient do what they want. If such patients can control their household appliances through a vBCI system, they can do small activities on their own and reduce their reliance on caregivers. Thus, in this study, we propose a BCI-based home care system (HCS). We hope that the HCS can help motor-disabled people improve their personal autonomy and self-reliance.

## 2. Materials and Methods

### 2.1. Subjects

The subjects used in this study were 15 healthy people (six females, aged 19–55), six motor-disabled people, and one man with ALS (SE7).  Table 2 summarizes the clinical data of the motor-disabled participants. All subjects were volunteers and had normal vision or vision corrected to normal, and they were without mental illness, head injuries, or drug treatments. No subjects underwent a training phase before the experimental procedure. Only subject E3 has experience in using an ERP-based visual BCI system. All subjects signed informed consent before participation in the study, which was approved by the National Cheng Kung University Human Research Ethics Committee. If a subject decided to quit during the experiment, we ended the experiment and deleted their data.

### 2.2. The vBCI-Based Home Care System (HCS)

#### 2.2.1. The Prototype of the HCS

**Figure 2:**
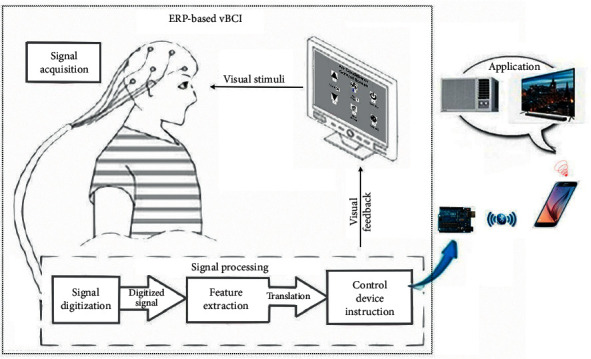
System architecture of the proposed HCS, including the ERP-based vBCI system and its applications.

In this study, the BCI module in the home care system is derived from the BCI module of our Chinese spelling system [8].  Figure 2 shows the design of the essential process of the vBCI-based home care system. The prototype includes three parts: signal acquisition, signal processing, and signal application.

The proposed vBCI module of the prototype, such as other human-machine interface systems for communication or control, comprises input/output processes. The BCI module requires the input of signals gained from the user's brain waves through an EEG. The EEG device in this system, which contains 32 channels, uses a typical noninvasive method [24]. The positions of electrodes accorded with the international 10–20 location system [58], and the vBCI module obtained signals from electrode O1 [8].

**Figure 3:**
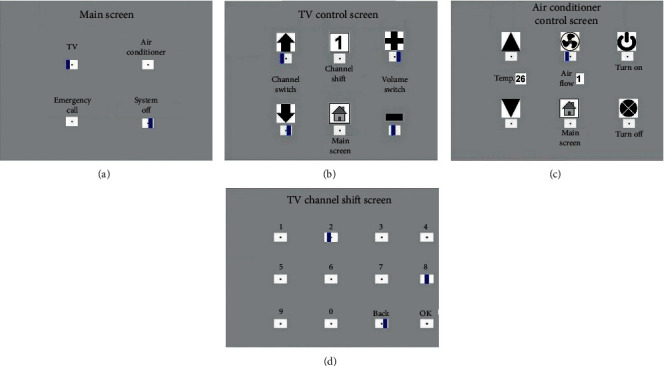
Four GUIs in the BCI module. (a) Main screen with four options; (b) TV control screen with six options; (c) air conditioner control screen with six options; and (d) TV channel shift screen with 12 options.

In all GUIs of the BCI module, there are several graphic options arranged in sequence on each GUI, as shown in Figure 3. Each option has a box, with a motion-strip as the visual stimulus, under it [25]. All motion-strips move from right to left to evoke ERPs, and the onset time of all motion-strips on the same GUI are inconsistent with each other [8]. Following the recommendations of the U.S. Department of Labor Occupational Safety and Health Administration, we let users sit 60∼80 cm away from the screen to reduce fatigue and eye strain [59]. The subject has to stare at the motion-strip of their choice during each trial. At the same time, the vBCI module obtains signals from the user's brain waves to gain the available ERPs components, including N200 and P300 [8, 60, 61]. Thus, the vBCI module can distinguish what the user wants and, then, output the control signal to the user's smartphone.

The vBCI module outputs communication signals to the user's smartphone via Arduino and the HC-5 Bluetooth module. The communication signals first execute an app on the user's smartphone designated ICAI1101, an application developed by the author. ICAI1101, then, triggers the corresponding app to make an emergency call or to send an IR signal to control a household appliance.

#### 2.2.2. Brain-Computer Interface for Stimulation

In this study, there are four GUIs in the BCI module, including the main screen, TV control screen, air conditioner control screen, and TV channel shift screen, as shown in Figure 3. Blue strips in the white box below each option on the GUIs of Figure 3 represent the motion-strip stimuli. In a single trial, all stimulus strips on the GUI move quickly from right to left six times. The onset time of the stimulus strips on the same GUI are asynchronous with respect to each other. Thus, the vBCI module reacts more rapidly and achieves higher accuracy [8]. Moreover, there is some complexity in the interface in most vBCI systems [22, 56, 60]. In this study, except for the TV channel shift screen, there are only four or six options on the other three screens to provide a faster, more intuitive, and friendlier interface for end-users.

#### 2.2.3. Smartphone Interface Design for Caregivers

**Figure 4:**
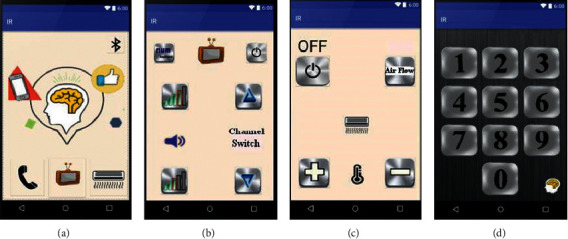
The caregiver GUIs of the ICAI1101 smartphone app: (a) main app screen on the smartphone; (b) TV remote controller; (c) air conditioner remote controller; and (d) TV channel shift screen.

When the BCI module identifies the option the user wants, it will send a command signal to the user's smartphone to control ICAI1101. Then, the GUI of the user's smartphone changes based on the user's selection. If there is a caregiver around the user, they can follow-up on the user's demands and help the user by using the GUI of the user's smartphone directly.  Figure 4 shows the app interfaces on the user's smartphone.

#### 2.2.4. Stimulation Trials

**Figure 5:**
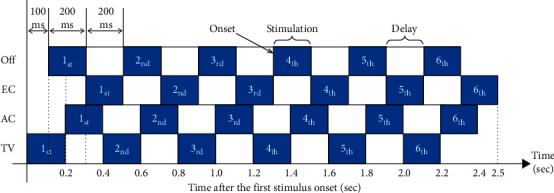
A stimulation schematic of one trial for the four options on the main screen. There are six instances of stimulation for every option in a single trial.

In this study, a stimulus is defined as the motion-strip of an option moving from right to left once, about 200 ms, as shown in Figure 3. The interval between two stimulus onsets in the same option was 200 ms. The stimulus onset asynchrony (SOA) between the two options was 50∼100 ms, depending on the number of options on the same GUI. SOA prevents stimuli from interfering with each other and shortens all stimulation time. In each trial, each option performs six stimuli in a regular sequence.  Figure 5 shows the stimulation sequence in a trial on the main screen. Thus, the time of 1 trial is roughly 3.0–3.8 sec (including roughly 500 ms for the system response), and the system can output the user's choice to the application immediately.

#### 2.2.5. The ERP Features of a Trial

**Figure 6:**
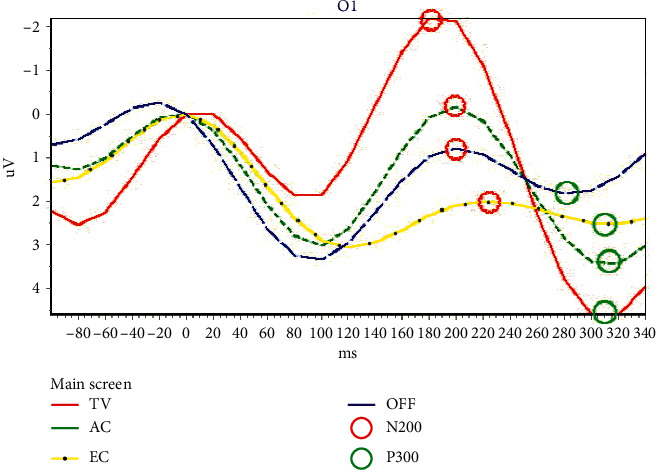
The ERPs of four options on the main screen from the output of the first trial of SE3. The red circle indicates the potential of N200, while the green circle represents the potential of P300.

In Figure 3(a), there are four options on the main screen. If the user stared at the motion-strip of the TV option, then the final ERP figure of this trial resembled Figure 6. The option with the red curve (TV) has the highest event-related potential for the N2P3 component. Thus, it is the target option and is the one (TV control) that the user selected and wanted to execute.

In Figure 6, the positions marked with red circles are the N200 troughs of the waveforms, and green circles indicate the P300 peak. The N200 potential has the smallest ERP value within 150 ms∼250 ms, while the P300 potential has the highest ERP value within 250 ms∼350 ms. The N2P3 value of one option is its P300 potential minus its N200 potential.

### 2.3. Experimental Procedure

#### 2.3.1. Overview of the Experimental Procedure

Each subject took about 0.5 to 1 hour to complete the experiment, depending on the accuracy of the trials.  Figure 2 shows the experimental setup. Before the test, the subject sat in front of the computer screen at a distance of roughly 80 cm. The procedure included four steps: (1) attaching the electrodes and checking the signals- 10 mins; (2) illustrating the experimental scheme and performing two trial runs as practice- 10 mins; (3) running the experimental procedure- 5∼35 mins; and (4) removing the electrodes and cleaning up- 5 mins.

The first step of the experiment was to attach electrodes to the subject's scalp and check the signals. The BCI module, then, connects with the user's smartphone via its Bluetooth module. Next, each subject performs 15 trials during the experimental procedure. In each trial, the user must choose an option from the GUI designed for the BCI module on the computer screen.

In each test, the subject had to gaze at the blue motion-strip of the option they wanted to choose. The system, then, collected the ERPs of all options available on the GUI from the EEG. After that, the BCI module identified the highest potential from the ERP components N200, P300, or N2P3. The option with the highest N2P3 potential should be the one the user was gazing at during the trial. The BCI module then sent a command signal to the user's smartphone via Bluetooth to make an emergency call or to control a household appliance via IR.

#### 2.3.2. Flowchart of the HCS Operating Procedures

**Figure 7:**
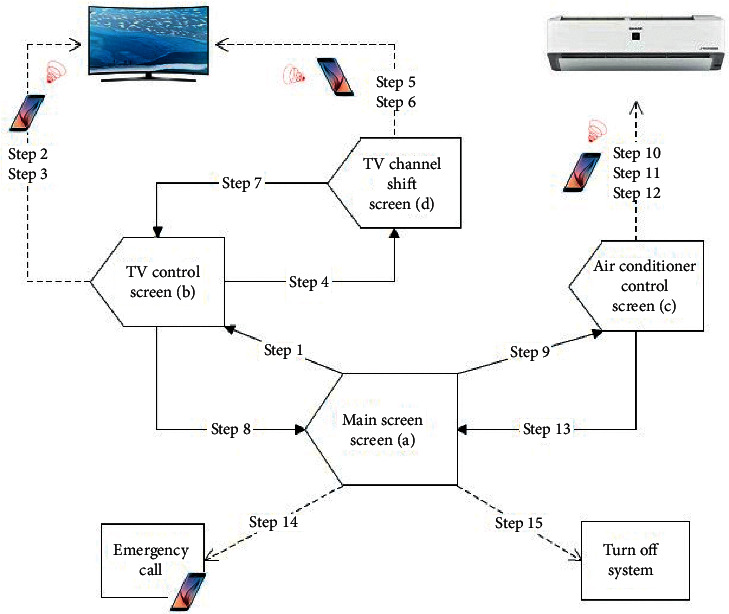
Flowchart of the vBCI operating procedures. The solid arrow line shows that the system sends an instruction to switch the screen of the system to the target GUI. The dotted arrow line shows that the system is only sending a command to do something, and the screen remains on the same GUI.

Figure 7 shows a flowchart of the BCI module operation. Each subject had to make 15 selections in the experimental procedure, representing 15 trials. In Figure 7, step 1∼step 15 represent the trial sequences.

Each subject performed their experimental procedure using the four GUIs. The TV and the air conditioner were under control during the process. They also made an emergency call before the end of the procedure.  Table 3 shows the details of the trial sequence. If the command given by the system is correct during a trial, the subject performs the following step of the procedure directly. Otherwise, the tester will discuss the cause of the error with the user and help the user return to the previous trial and try again.

### 2.4. Experimental Setup

#### 2.4.1. Experiment Equipment

The equipment used to acquire the EEG data included a 32-channel EEG amplifier, an ISO-1032CE, and the control unit, CONTROL-1132, produced by Braintronics B.V. Company. The system uses PCI-1713 to convert analog data to digital data. The authors wrote the vBCI module using Borland C++ Builder and wrote the ICAI1101 smartphone app in Java. The BCI module used an Arduino Uno and HC-05 Bluetooth module to communicate with the user's mobile phone.

#### 2.4.2. Data Acquisition

In the EEG acquisition settings, the sampling rate is 500 Hz, and the impedance remains below 10 kΩ. The EEG acquires the subject's brain waves from electrode O1 on the user's scalp. The electrodes for eye movement detection are FP1 and FP2. The reference electrodes are A1 and A2. The ground electrode is FPz [8]. All electrodes are wet Ag/AgCl electrodes. The EEG amplifier (ISO-1032CE) amplifies and records the potentials of the user's brain waves. The data are filtered with a 0.3∼15 Hz band-pass filter in the control unit, CONTROL-1132. Then, PCI-1713 converts the analog data to digital data and sends the data to the vBCI module for interpretation.

#### 2.4.3. Data Processing

(1). ERPs acquisition: in each trial, each motion-strip moves from right to left six times, as shown in Figure 5. The system records the time of onset for each motion-strip and obtains the brain waves of the subjects. The system, then, segments the EEG into ERPs within intervals ranging from‒100 ms to 800 ms of the onset time. Thus, there are six segments for each option. The system, then, sums the six ERP segments of each option and averages them to obtain the final ERPs for each option.

(2). ERPs analysis: the system saves and analyses the final ERPs for each option in each trial. The system, thus, finds the N200 value and the P300 value of each option and, then, determines the N2P3 value. Next, the system compares the N2P3 value of all options to each other to identify which option the user selected.

(3). Instruction output: the system translates the ERP analysis results into a BCI instruction and sends it to the user's smartphone via Bluetooth. When the smartphone receives a BCI instruction, it runs the application to make an emergency call or to control an appliance via IR.

### 2.5. System Assessment with Bit Rate

In addition to the accuracy rate, the rate at which information per unit of time is obtained is particularly important for evaluating a BCI system. To calculate the number of bits available per minute, the bit-rate calculation in this study uses the definition of Wolpaw [62], as follows:

(1) $$\begin{array}{c} \text{bit}-\text{rate}=M\left\{\log_{2}\;N+P\;\log_{2}\;P+\left(1-P\right)\log_{2}\left[\frac{\left(1-P\right)}{\left(N-1\right)}\right]\right\}, \end{array}$$

where *M* indicates the number of choices made in a minute, N is the number of options, and P is the accuracy rate.

## 3. Results and Discussion

Table 2 shows information about all the subjects who participated in the experiment. Although the participants could ask to stop the procedure at any time, no one did. The system used the N2P3 component to interpret the output of the EEG online. However, to find the optimal solution, we analyzed the features of ERP components N200, P300, and N2P3 of all users offline. The experimental results were analyzed as follows.

### 3.1. Discriminating Features of ERPs

**Figure 8:**
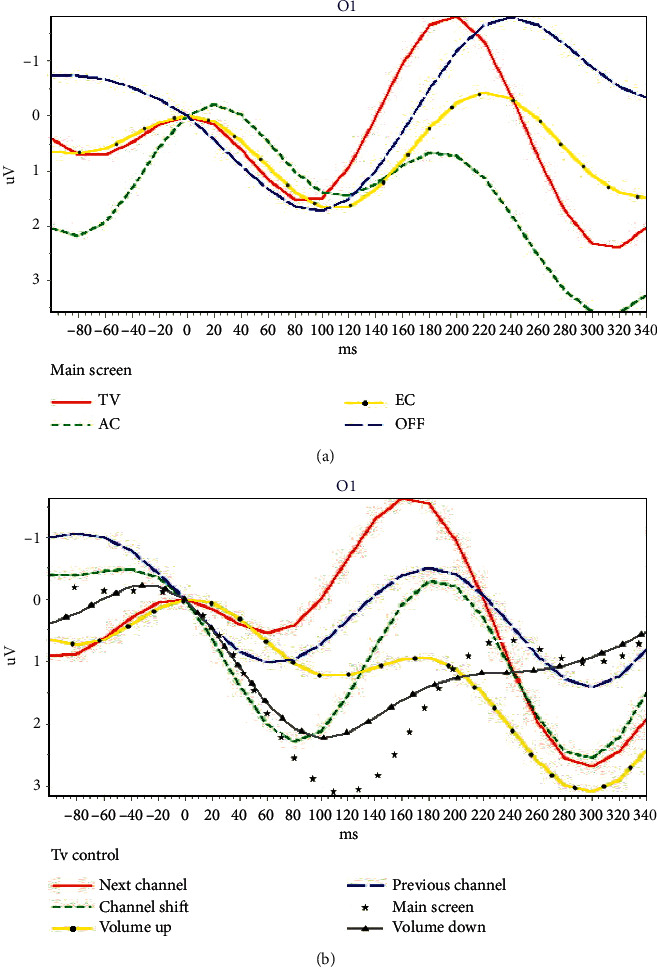
Two ERP samples from one motor-disabled subject. (a) The ERPs from the main screen of the BCI system (4 options); (b) the ERPs from TV control screen of the BCI system (6 options).

Figure 8 illustrates the discriminating features of ERPs.  Figure 8(a) is the output figure obtained from the 1^st^ trial of E14, while Figure 8(b) is the output figure from the 3^rd^ trial of SE1.

In Figure 8(a), the red curve represents enter the TV control screen, the dotted green curve represents enter the air conditioner control screen, the dotted yellow curve represents making an emergency call, and the blue segment curve represents shut down the system.

In Figure 8(b), the red curve represents change to the next channel, the dotted green curve represents enter the TV channel shift screen, and the dotted yellow curve represents increasing the volume. The blue segmented curve is a change to the previous channel, the dotted white curve represents backing to the main screen, and the dotted gray curve represents decreasing the volume.

Figure 8(a) shows that the N200 value (−1.7969 *µ*V) of the solid red curve (TV option) on the main screen is the lowest ERP, and P300 value (3.5418 *µ*V) of the dotted green curve (AC option) is the highest ERP.  Table 4 shows that the maximum ERP of N2P3 (4.0814 *µ*V) is obtained from the solid red curve (TV option). Thus, the user wanted to access the function of the TV control during the online experimental process. This result complies with the requirements of the experimental procedure. The offline analysis shows that the result is correct if using N200 for interpretation. However, the result when using P300 for interpretation, AC, is wrong.

Figure 8(b) shows that the N200 value (−1.6458 *µ*V) of the solid red curve (Next Channel) on the TV screen is the lowest ERP, and P300 value (3.0962 *µ*V) of the dotted yellow curve (volume up) is the highest ERP.  Table 5 shows that the maximum ERP of N2P3 (4.3471 *µ*V) gains from the solid red curve (next channel). Thus, it means the user wanted to access the function of the next channel during the online experimental process. This result complies with the requirements of the experimental procedure. The offline analysis shows that the result, the solid red curve (next channel), is right if using N200 for interpretation. However, the result obtained when using P300 for interpretation, volume up, is wrong.

### 3.2. Experimental Results for Healthy Subjects

#### 3.2.1. Accuracy and Bit-Rate Analysis

**Figure 9:**
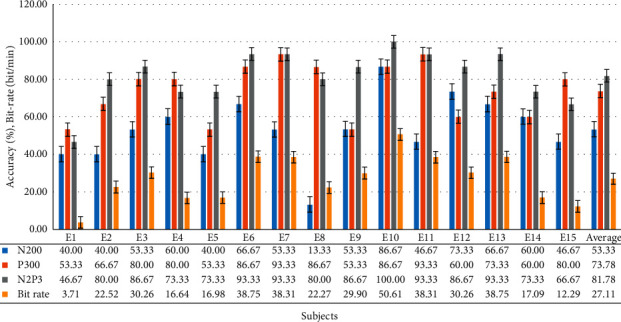
The accuracy levels and bit rate attained by all 15 healthy subjects.

Fifteen healthy subjects participated in the experiment. The experimental results showed that the feature of N2P3 enabled the best discrimination. The average accuracy across all 15 users was 81.78%, meaning that of all 15 commands, 12 were performed right. The precision attained by 10 of the 15 subjects was greater than 80%. The accuracy of E10 was even 100%. However, the accuracy of E1 and E15 was unacceptable. These two subjects may not be able to adapt to the BCI system or were disturbed by other factors, resulting in reduced efficiency.  Figure 9 summarizes the accuracy levels and bit rate acquired by the 15 healthy subjects.

The average bit rate attained by all 15 healthy subjects is 27.11. It is better than that of other studies [25, 62, 63].

#### 3.2.2. Analysis of Sum of Correct Choices Made by All Healthy Subjects in Each Trial

**Figure 10:**
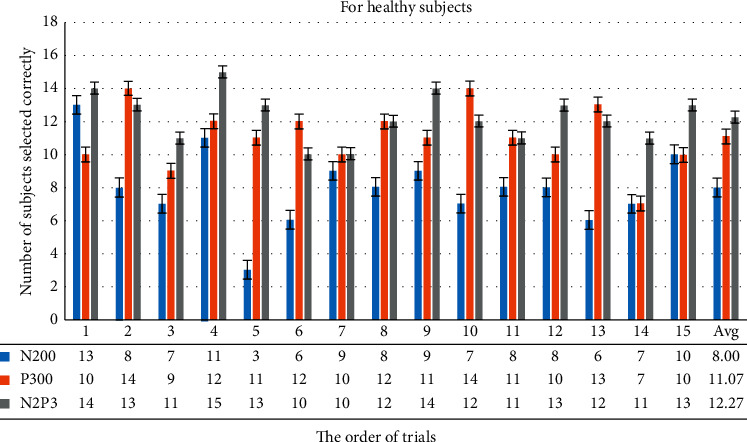
This bar chart summarizes the number of correct selections for each healthy subject for each trial. For example, if the system uses N2P3 to interpret the EEG, the choices from 14 of all 15 users are correct in the first trial, while N200 13 are correct, and only 10 are correct for P300.

Figure 10 shows that if the system uses N2P3 to interpret the EEG, the average number of correct selections for each healthy subject was 12.27 on average, while N200 it is only 8 and 11.07 for P300. It is important to note that the 6^th^ and 7^th^ trials exhibited lower online performance with the system choosing the right option for only 10 of 15 users (66.67%). By contrast, all the subjects chose the correct option on the 4^th^ trial, and the number of correct selections in 10 of all 15 trials is greater than 12 (80%). Thus, using N2P3 for interpretation is the optimal solution.

#### 3.2.3. The Paired-Sample t-Test Analysis of the Results of the Targeted Option vs. Nontargeted Options

Table 6 shows the paired-sample t-test analysis of the ERP components of the target option and the nontarget options. The results show that the features of the ERP components, P300 and N2P3, can be used as discriminating features. However, there was a significant difference in the results between N2P3-targeted and P300-targeted. Thus, using the component N2P3 for interpretation is the optimal solution.

### 3.3. Experimental Results for Motor-Disabled People and ALS

#### 3.3.1. Accuracy and Bit-Rate Analysis

**Figure 11:**
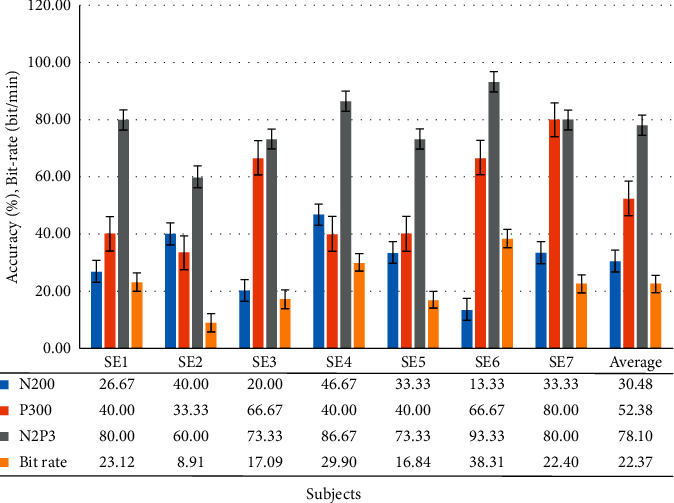
Accuracy levels and bit rate attained by all motor-disabled subjects (including one ALS, SE7).

Six motor-disabled people and one man with ALS participated in the experiment. The experimental results showed that the feature of N2P3 offered the best discrimination. The average accuracy across all seven users was 78.10%, meaning that of all 15 commands, 11 were performed right. However, the accuracy of SE2 is not acceptable. This subject may not be able to adapt to the BCI system or was disturbed by other factors, resulting in reduced efficiency.  Figure 11 summarizes the accuracy levels acquired by the seven with physical disabilities.

The average bit rate attained by all seven disabled subjects is 22.37. Although the average bit-rate attained by the disabled group is lower than that of the healthy group, it is also better than that of other studies [25, 62, 63].

#### 3.3.2. Analysis of Sum of Correct Choices Made by All Motor-Disabled Subjects in Each Trial

**Figure 12:**
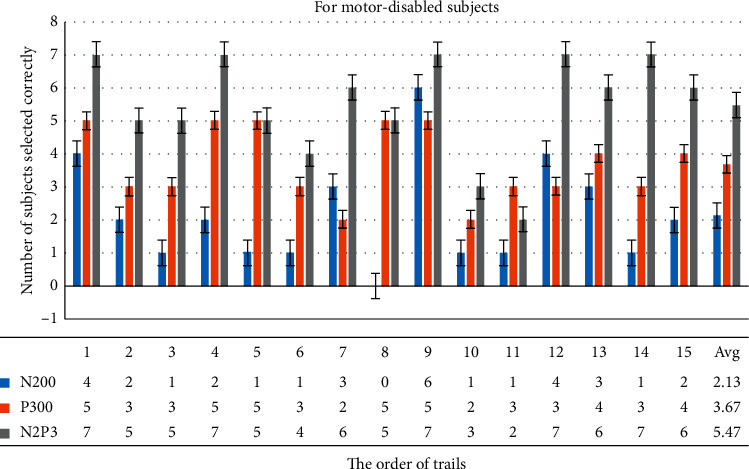
This chart summarizes the correct selections of all motor-disabled subjects for each trial. For example, if the system uses N2P3 to interpret the EEG, the choices from 7 of 7 users are correct in the first trial, while 4 are correct with N200 and 5 with P300.

Figure 12 shows that if the system uses N2P3 to interpret the EEG, the average number of disabled subjects selecting correctly is 5.47, 2.13 for N200 and 3.67 for P300. It is important to note that the 10^th^ and 11^th^ trials exhibited lower performance online. By contrast, all the subjects chose the correct option in the 1^st^, 4^th^, 9^th^, 12^th^, and 14^th^ trials. Thus, using N2P3 for interpretation is the optimal solution for motor-disabled subjects. These results are the same as those of the group of healthy subjects.

#### 3.3.3. The Paired-Sample *t*-Test Analysis of the Results of the Targeted Option vs. Nontargeted Options

Table 7 shows the paired-sample *t*-test analysis of the ERP components of the target option and the nontarget options. Although the *p*-value (.004) of case N200 is less than 0.01, Figure 11 shows that the average accuracy is 30.48% if the system used the component N200 for interpretation. Thus, only N2P3 can be used as the discriminating feature for motor-disabled subjects.

### 3.4. Independent-Sample *t*-Test for the Results for the Healthy Subjects and the Motor-Disabled Subjects (including One ALS)

In this study, we conducted experiments with 15 healthy subjects, six motor-disabled subjects, and one ALS. The average accuracy attained by the 15 healthy subjects was 81.78% if using N2P3 (online) for interpretation, while the average accuracy attained by the seven motor-disabled subjects was 78.10%. The disabled group has a lower accuracy level than the healthy group. However, both groups had an average accuracy of more than 75%.

We compared the results of these two independent samples, as shown in Table 8. The results show that if the system used the N200 or P300 component for interpretation, there was a significant difference in the results between the healthy group and the disabled group. That is, the system may not be acceptable for disabled people. Yet, when the system uses N2P3 for interpretation, there is no significant difference between the two groups (*t* = 0.6258, *p*=0.5385). Thus, the proposed vBCI system appears to be suitable for our end-user, motor-disabled people, when the system uses N2P3 for interpretation online. It enables the HCS to reach a desirable level.

The average bit rate attained by all 15 healthy subjects is 27.11 (Figure 9). The average bit rate attained by all seven disabled subjects is 22.37 (Figure 11). Although the average bit rate attained by the disabled group is lower than the average bit rate attained by the healthy group, the difference is not significant (*t* = 0.8793, *p*=0.3897).

## 4. Discussion

The aim of the present study is two-fold: (1) to develop a BCI-based home care system to help end-users control their household appliances and (2) to assess whether the architecture of the HCS is easy for motor-disabled people to use.

First, we designed and developed a BCI-based home care system (HCS). We designed the HCS to make an emergency call or control the household appliances, such as TV and air conditioner, via a smartphone. Thus, end-users can improve personal autonomy and reduce their dependence on caregivers.

Second, most previous research has not experimented with end-users. Thus, the second purpose of this study was to assess the usefulness of the system with motor-disabled subjects. We conducted experiments with both healthy and motor-disabled subjects. One subject had ALS.

Previous researchers attempted to improve the performance of their BCI systems [7, 9, 21, 23, 60]. Thus, improving the accuracy of the HCS is the primary mission of our study. To improve the efficiency of HCS, we adjusted three details: the number of electrodes, the brain-computer interface, and the method the system uses to interpret the ERPs.

Most BCI studies use Fz, Cz, Pz, Oz, and other electrodes to collect data [9, 22, 23, 29, 34, 45, 60]. Of these, Zhang et al. also used the O1 and O2 electrodes to obtain data [10]. Based on the previous research results of our laboratory, the ERPs acquired from electrodes O1 and O2 yield outstanding accuracy and are better than ERPs from other electrodes when we asked the user to stare at the GUI [8, 26]. Furthermore, the fewer the electrodes are, the more comfortable the user is. Thus, the system used electrode O1 only to obtain the ERP data in this study.

To use an electrode, Q1, to gain the data, we ask the user to stare at the GUI when using the system. The system asynchronously shows the stimuli to shorten stimulation times. In the HCS there are four options on the main screen, six options on the TV and AC control screen, and 12 options on the TV channel shift screen. Figures 10 and 12 show that the number of options on the GUI has nothing to do with the number of users correctly selecting in each trial. The average bit rate in both groups was better than that of related studies. Although the average bit rate attained by the disabled group is lower than that attained by the healthy group, the difference is not significant (*t* = 0.8793, *p*=0.3897). Therefore, we inferred that the interface of the HCS is also applicable to motor-disabled people.

Previous studies have stated that component P300 provided an excellent level of discrimination [29, 60, 61]. However, in earlier studies in our laboratory, component N2P3 presented the best level of discrimination [8]. Thus, we compared ERP components N200, P300, and N2P3 to determine what the best feature for discrimination is.

When using component P300, the healthy and the motor-disabled subjects had an average efficiency of 73.78% (SD = 14.79) and 52.38% (SD = 18.23), respectively. Although the precision attained by 8 of the 15 healthy subjects was greater than 80%, only SE7 (ALS) obtained an accuracy of 80% when using P300 for interpretation. However, for the online experimental results (using component N2P3), the healthy and the motor-disabled subjects exhibited an average efficiency of 81.78% (SD = 13.69) and 78.10% (SD = 10.69), respectively. The precision attained by 10 of the 15 healthy subjects was greater than 80%, and the precision obtained by three of the seven motor-disabled subjects was greater than 80%. Furthermore, Figure 11 shows that, from SE1 to SE6, the accuracy using N2P3 for interpretation is the best. SE7 (ALS) had an accuracy of 80% using component N2P3 and again using P300.

Although the disabled group has a lower accuracy level than the healthy group, the difference is not significant (*t* = 0.6258, *p*=0.5385). The average accuracy attained by the seven motor-disabled subjects was 78.10%, more than the 75% when using N2P3 for interpretation. To this point, these results are consistent with those of Huang [8] which show that the ERP component N2P3 is the optimal solution for discrimination in the HCS. Thus, the HCS is suitable for end-users, including motor-disabled people. The HSC proposed in this study reaches a desirable level of performance when the system uses N2P3 for interpretation.

The second issue in system construction is making the system easy for end-users. This question included two key points: whether the GUI of the HCS is friendly and whether the remote controls for all appliances can be integrated into one remote control.

First, Figure 10 shows that more than 12 subjects made the right selections in 10 of 15 trials (over 80%). Figure 12 shows that more than six subjects made the right selections in 8 of 15 trials (over 85%). Most trials exhibited a high correct selection rate. Coupled with speedy bit rate, we reason that the interface in the system is easy to use.

Second, there are often two common household appliances, TVs and air conditioners, in the same room. Every home appliance has a dedicated remote control. If all home appliances can share the same remote control, the system can be easy for end-users to use. In this study, we added a smartphone to the HCS. A smartphone with IR communication technology may act as a remote control. It can emit a distinct IR wavelength to control any household appliance. Thus, we developed an app called ICAI1101 to control the TV and air conditioner using a smartphone via IR. Such a BCI system would make it easier for the end-user to control their home appliances.

ICAI1101 is first installed on a smartphone. When the user makes a choice during use, ICAI1101 can act following the user's choice. Furthermore, ICAI1101 not only makes emergency calls and controls the TV or air conditioner but can also integrate other apps. The rapid growth of the Internet and the popularity of smartphones have had an immense impact on human life in the last two decades [22]. Innumerable apps designed for smartphones reside in mobile app stores. Millions of apps are aimed at motor-disabled people. ICAI1101 can easily integrate these apps into the BCI system. In the future, we plan to integrate other apps into the BCI system. Thus, the HCS could help end-users to learn and communicate with others.

Tables 3 and 5 show that the accuracy of E1, E15, and SE2 is not acceptable. These subjects may have individual factors that resulted in lower efficiency. Development of the subsequent vBCI system should address the aforementioned personal questions. This will allow the HCS to help end-users achieve better quality of life.

## 5. Conclusions

In this study, we have proposed a home care system that combines BCI with a smartphone. The HCS helps end-users, motor-disabled people, make an emergency call or control their household appliances. Thus, end-users can take care of themselves with only eye muscle movement. Fifteen healthy subjects and seven motor-disabled subjects (including one with ALS) participated in clinical trials. Because of the high accuracy-rate and rapid response of the system during the online experimentation, most subjects of both groups can rapidly complete the experimental procedure in less than the preset time, 35 minutes. In the offline analytics, the data collected enabled us to evaluate and improve the performance of the system. The results showed that the disabled group has a lower accuracy level than that of the healthy group, but the difference is not statistically significant. The average accuracy of the disabled group (78.10%) not only exceeded the chance level but was also higher than 75%. The bit-rate analysis yielded conclusions similar to those of the accuracy analysis. Thus, when a user chooses an option, the accuracy of the target option in a short period exceeds three-fourths. We, therefore, reason that the HCS is a viable system for motor-disabled people.

The HCS is a system that can be used without prior training. The bit rate of the HCS is close to that of a previous study, the Chinese Spelling System, performed in our Lab [8], and is better than that of other studies [25, 62, 63]. Such a fast bit rate and high accuracy rate make the HCS easy to use. Even if the user selects the wrong option, the system can be returned to the correct position in a short time by reselection. More importantly, only one electrode, O1, is required to measure the EEG signals, enabling the HCS to have good usability in practical use. Thus, we confirmed the feasibility and practicability of this home care system approach.

## Figures and Tables

**Table 1:** Recent studies of BCI-based systems implemented in real-world scenarios.

Study	Main function	Stimulation modality	Electrodes	Subjects	Accuracy (%)	Bit rate
[25]	Speller	ERP: motion-onset-P300	Fz, Cz, Pz, Oz, P7, and P8	10 CS	N2-91.5, P3-72.4	N2-15.91, P3-12.84

[8]	Chinese speller	ERP: motion-onset-N2P3	F3, F4, C3, C4, P3, P4, O1, O2, Fz, Cz, and Pz	7 CS	80% using O1 only	27.8

[9]	Speller	ERP + SSVER RC paradigm	Cz, Pz, P3, P4, O1, O2, POz, PO7, and PO8	14 CS	After 8 trials: >95	53.6

[26]	Robot control	ERP: motion-onset-N2P3	O1	12 CS	80% using O1 only	353.33 s for 26.33 comm.

[7]	Robot control	EOG + EEG: flash on eight direct	Fz, Cz, Pz, Oz, P7, P3, P4, and P8	13 CS	After 5 trials:>99.04	—

[21]	Speller	ERP + SSVER RC paradigm	Fz, Cz, Pz, P3, P4, PO7, PO8, POz, Oz, O1, and O2	13 CS	95.18 for hybrid	50.41 for hybrid

[23]	Healthcare BCI syst.	ERP + SSVER RC paradigm	Cz, Pz, O1, O2, and Oz	5 CS	ERP: 95.5SSVER:93	—

[10]	Environmental control	ERP-P300RC paradigm	Fz, FCz, Cz, CPz, P7, P3, Pz, P4, P8, O1, Oz, and O2	6 MDS, 2 CS	89.6	734.3 s for 30 comm.

[22]	Use of social networks	ERP-P300 RC paradigm	Fz, Cz, Pz, P3, P4, PO7, PO8, and Oz	18 MDS, 10 CS	80.6 for MDS, 92.3 for CS	1.47 OCM for MDS, 2.06 for CS

RC paradigm: the row-col paradigm; “N” indicates the number of subjects; “CS” stands for control abled subjects; “N2” stands for N200 evoked potential; “P3” stands for P300 evoked potential.

**Table 2:** Clinical data of the motor-disabled participants.

Subject	Age	Gender	DD	Disease
SE1	35	M	Moderate	Spinal cord injury
SE2	37	M	Moderate	Tetraplegia
SE3	46	M	Moderate	Spinal cord injury
SE4	42	M	Mild	Spinal cord injury
SE5	39	M	Moderate	Spinal cord injury
SE6	43	M	Mild	Spinal cord injury
SE7	50	M	Marked	ALS

**Table 3:** Details of the 15 trial sequences.

Step	1	2	3	4	5	6	7	8	9	10	11	12	13	14	15
GUI	Main screen	TV screen	TV screen	TV screen	TV channel shift	TV channel shift	TV channel shift	TV screen	Main screen	Air condi.	Air condi.	Air condi.	Air condi.	Main screen	Main screen

Option to choose	Enter TV	Volume up	Next channel	Enter channel shift	Choose channel 1	Choose OK	Back TV	Back to main	To air condi.	Turn on AC	Temp down	Air Flow	Back to main	Emergency call	System off

**Table 4:** The ERP values (*µ*V) of all options in the main screen obtained from the first trial of subject E14.

Options	N200	P300	N2P3	Result	
				Online	Offline
TV	−1.7969	2.2845	4.0814	✓	N200, N2P3
AC	0.6518	3.5418	2.8900		P300
EC	0.0030	1.0302	1.0272		
Off	−1.7847	−0.9263	0.8584		

**Table 5:** The ERP values (*µ*V) of all options in the main screen obtained from the 3^rd^ trial of subject SE1.

Options	N200	P300	N2P3	Result	
				Online	Offline
Next channel	−1.6458	2.7013	4.3471	✓	N200, N2P3
Channel shift	−0.3196	2.5717	2.8913		
Volume up	0.9145	3.0962	2.1817		P300
Prev. channel	−0.5131	1.4089	1.9220		
Main screen	0.8788	1.0479	0.1691		
Volume down	1.3293	1.1093	−0.2200		

**Table 6:** Paired-sample t-test results of all trials for the 15 healthy subjects. *α* = 0.01, *N* = 225.

Case		*T* value	*p* value
N200	Targeted vs. nontargeted	0.747	0.467
P300	Targeted vs. nontargeted	6.225	0.000^*∗∗∗*^
N2P3	Targeted vs. nontargeted	8.998	0.000^*∗∗∗*^
N2P3 vs. P300	N2P3-targeted vs. P300-targeted	2.276	0.039^*∗*^

^*∗*^ *p* < 0.05; ^*∗∗*^*p* < 0.01; ^*∗∗∗*^*p* < 0.001.

**Table 7:** Paired-sample *t*-test results of all trials for the seven motor-disabled subjects. *α* = 0.01, *N* = 105.

Case		*T* value	*p* value
N200	Targeted vs. nontargeted	−4.509	0.004^*∗∗*^
P300	Targeted vs. nontargeted	0.346	0.741
N2P3	Targeted vs. nontargeted	6.953	0.000^*∗∗∗*^

^*∗∗*^ *p* < 0.01; ^*∗∗∗*^*p* < 0.001.

**Table 8:** Independent-sample *t*-test of the accuracy of the healthy subjects and of the motor-disabled subjects, *α* = 0.01.

Case		*F*-test *p* value	*T* value	*p* value
N200 targeted	Healthy vs. disabled	0.1596	3.1693	0.0048^*∗∗*^

P300 targeted	Healthy vs. disabled	0.2428	2.9396	0.0081^*∗∗*^

N2P3 targeted	Healthy vs. disabled	0.2817	0.6258	0.5385

Bit rate	Healthy vs. disabled	0.2576	0.8793	0.3897

^*∗∗*^ *p* < 0.01; ^*∗∗∗*^*p*  <  0.001.

## Data Availability

Data are available on request. E-mail: klhsieh@gmail.com.
